# Métastases gastro-intestinales du cancer du sein: à propos de 2 cas

**DOI:** 10.11604/pamj.2013.15.74.2885

**Published:** 2013-06-25

**Authors:** Mezouar Loubna, El Hfid Mohamed, El Harroudi Tijani, Ghadouani Fouzia, Haj Kacem Hanane, Bourhaleb Zouhour, Ouabdelmoumen Asmae

**Affiliations:** 1 Faculté de Médecine et de Pharmacie, Université Mohammed Premier, Maroc; 2 Centre régional d'oncologie Hassan-2-Oujda, Maroc; 3 Département de radiologie, Centre Hospitalier Régional El Farabi, Oujda, Maroc

**Keywords:** Cancer du sein, métastases gastrique, métastases duodénales, Breast cancer, gastric metastases, duodenal metastasis

## Abstract

Le cancer du sein est le cancer le plus fréquent chez la femme, notamment au Maroc, avec un taux de mortalité élevé. Les métastases gastro-intestinales d'un carcinome canalaire du sein sont rares. Leur diagnostic est difficile du fait de la nature non spécifique des symptômes. Nous rapportons deux observations originales de métastases gastroduodénales d'un cancer canalaire infiltrant du sein. Les métastases gastro-intestinales du cancer du sein sont très rares; la présence de symptômes gastro-intestinaux chez une malade ayant un antécédent de cancer du sein doit faire suspecter une atteinte métastatique gastro-intestinale.

## Introduction

Le cancer du sein est le cancer le plus fréquent chez la femme, notamment au Maroc, avec un taux de mortalité élevé. Le Carcinome canalaire infiltrant du sein est pourvoyeur de métastases osseuses, hépatiques, pulmonaires et cérébrales alors que le carcinome lobulaire est plutôt à l'origine de métastases gastro-intestinales, gynécologiques et péritonéales. Les métastases gastro-intestinales d'un carcinome canalaire du sein sont rares. Leur diagnostic est difficile du fait de la nature non spécifique des symptômes. Nous rapportons deux observations originales de métastases gastroduodénales d'un cancer canalaire infiltrant du sein.

## Patient et observation

### Observation 1

**Figure 1:**
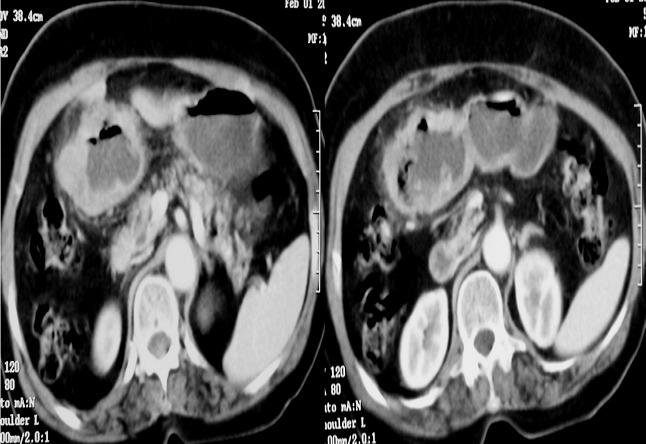
Coupes scannographiques axiales réalisées après injection du produit de contraste montrant un processus tumoral pariétal circonférentiel gastrique pylorique avec infiltration de la graisse péri tumorale

Patiente âgée de 70 ans, traitée en 2008 pour un carcinome canalaire infiltrant du sein droit classé initialement T4N2M0 n'exprimant pas les récepteurs hormonaux oestrogéniques ni progesteroniques. Un bilan d'extension à la recherche des métastases à distance s'est révélé négatif. La patiente a bénéficié d'une chimiothérapie néo adjuvante (type FEC) avec une bonne réponse clinique et disparition des signes inflammatoires après la troisième cure de chimiothérapie. Le traitement a été complété par une chirurgie type mastectomie avec un curage axillaire. L'étude anatomo-pathologique a montré, à la macroscopie, une tumeur de 2 cm associée à 11 ganglions. A la microscopie, il s'agissait d'un carcinome canalaire infiltrant de grade III avec images d'emboles vasculaires. Neuf ganglions sur les 11 prélevés étaient envahis. Une radiothérapie externe locorégionale a été délivrée à la dose de 50 Gy. Après un recul de sept mois de la fin du traitement, la patiente a présenté un épisode d'hématémèses. Une fibroscopie œsogastroduodénale a retrouvé une tumeur antrale pré pylorique. L’étude histologique de la biopsie antrale a permis de porter le diagnostic d'un adénocarcinome bien différencié de l'estomac avec une recherche des récepteurs hormonaux positifs (oestrogéniques et progesteroniques). Un scanner thoraco-abdominal a montré un processus tumoral antro-pylorique avec signe d'extension extra pariétale mais sans extension à distance (Figure 1). Vue l’état général de la patiente OMS2, on a démarré l'hormonothérapie à base du Tamoxiféne 20 mg. La patiente est décédée après 3 mois de traitement.

### Observation 2

**Figure 2:**
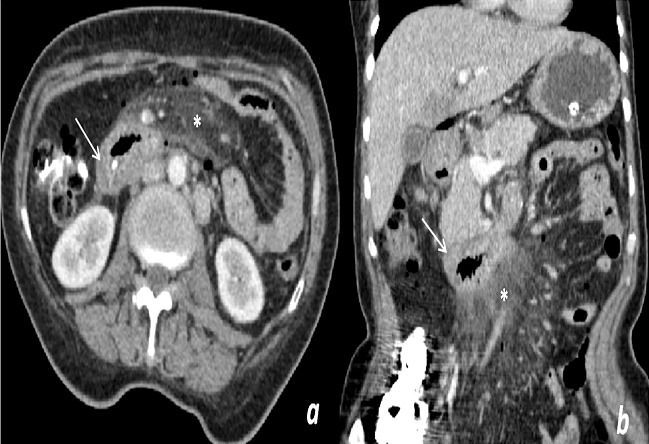
Coupe scannographique axiale (a) et coronale (b) réalisées après injection du produit de contraste iodé, montrant un épaississement pariétal tumoral du troisième duodénum (flèche) avec adénomégalies péri aortiques. A noter une adénolymphite mésentérique réactionnelle (*)

Patiente âgée de 50 ans ayant comme antécédent notable un carcinome canalaire infiltrant grade III SBR du sein droit, traitée en janvier 1996 par mastectomie avec curage axillaire droit. La tumeur était classée pT1cN0M0. La malade avait bénéficié de 6 cures de chimiothérapie type FAC suivie d'une hormonothérapie type Tamoxiféne 20 mg pendant 5 ans. En 2011, elle a présenté des vomissements avec apparition des poly adénopathies basi-cervicales gauches et perte de poids importante de 35Kg en six mois. Un transit du grêle a objectivé une dilatation duodénale secondaire à une image lacunaire sténosante d'allure tumorale au niveau de D3. Un scanner thoraco-abdominal a retrouvé un épaississement pariétal de D 3 à la première anse jéjunale manifestement tumoral, associé à une densification de la graisse et à de nombreuses adénomégalies (6-30mm) locorégionales et rétro-péritonéales sans lésion hépatique (Figure 2). A l’étage thoracique, des poly adénopathies sus claviculaires gauches (mesurant de 14 à 30 mm de petit axe) médiastinales (pré et sous carinaires et de la fenêtre aorto-pulmonaires nécrosées, mesurant 14 mm de petit axe) et un épanchement pleural liquidien bilatéral. La patiente a bénéficié d'une résection duodénale dont L'analyse histologique a mis en évidence un adénocarcinome moyennement différencié invasif infiltrant toute l’épaisseur de la paroi duodénale et arrivant au contact de la séreuse avec présence de nombreux emboles tumoraux intravasculaires. La paroi duodénale à distance de la tumeur ne présente pas d'anomalies.

**Figure 3:**
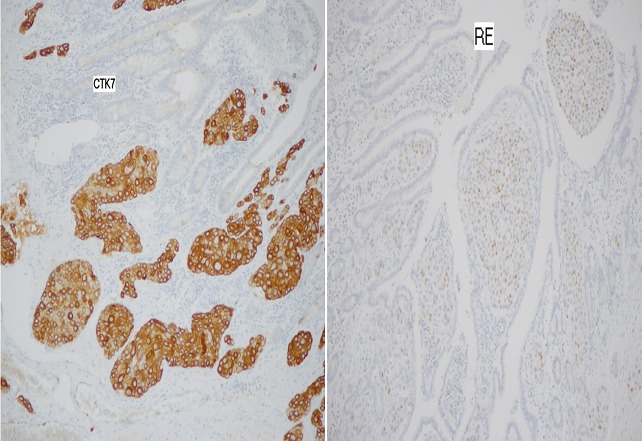
Les cellules tumorales expriment fortement la cytokératine 7, les récepteurs aux œstrogènes

En immunohistochimie, les cellules tumorales expriment fortement la cytokératine 7, les récepteurs aux œstrogènes (Figure 3) et Her-2-neu et n'expriment pas la cytokératine 20, les récepteurs à la progestérone, la chromogranine et le CD56. Devant l'aspect histologique et le profil immunohistochimique le diagnostic a été orienté vers une métastase duodénale d'un adénocarcinome mammaire connu chez cette patiente. On a démarré une chimiothérapie type paclitaxel et une thérapie ciblée type trastuzumab. L’évolution a été marquée par la disparition des adénopathies cervicales dés la deuxième cure. Dix- huit mois après le début du traitement, la patiente est toujours en rémission radiologique maintenue. Le scanner cervico-thoraco-abdominal, a montré l'absence de localisations secondaires hépatique ou pulmonaires et absences d'adénopathies cervicales.

## Discussion

Les métastases osseuses, pulmonaires et hépatiques du cancer du sein sont fréquentes, mais les métastases gastro-intestinales sont rares. Les métastases gastro-intestinales du cancer du sein sont le plus souvent non diagnostiquées et occasionnellement rapportées. Les études d'autopsie ont rapporté que la fréquence de métastase gastrique varie entre 7.4% et 18%, tandis qu'une étude clinique de 596 patients suivis pour métastases gastriques d'origine mammaire a décrit seulement une fréquence de 6% [1, 2]. Dans l'expérience de McLemore sur 12,001 patients suivis pour cancer du sein métastatique, seuls 73 patients ont développé des métastases gastro-intestinales [3]. Le carcinome lobulaire du sein est responsable de 80% des cas de métastases gastro-intestinales. En effet, dans l'expérience de Taal; incluant 51 patients suivis pour métastases gastrique d'origine mammaire; 70% des patients présentaient un carcinome lobulaire infiltrant du sein [4]. Alors que nos deux patientes étaient suivies pour CCI du sein. Ceci dit, le diagnostic des métastases gastro-intestinales d'origine mammaire est un diagnostic d’élimination. En effet, c'est un diagnostic difficile du fait de la nature non spécifique des symptômes et aucun élément paraclinique n'apporte de certitude diagnostique. Les données immuno-histochimiques peuvent orienter le diagnostic (expression des anticorps anticytokératine 7 et anti-estrogènes et négativité d'expression pour la cytokératine 20) [5]. Des modalités thérapeutiques comme le traitement hormonal, chimiothérapie et la thérapie ciblée représentent les meilleures options thérapeutiques dans la prise en charge des métastases Gastro-intestinales d'origine mammaire. Les indications chirurgicales sont réservées aux métastases uniques et aux situations d'urgence (hémorragie massive, obstruction persistante ou perforation) [6, 7]. Dans une étude de McLemore, sur 23 patients suivis pour des métastases gastro-intestinales d'origine mammaire, 12 ont bénéficié d'une chirurgie palliative non précisé avec une survie médiane de 44 mois versus 9 mois chez les patients non opérés [3]. Le pronostic des métastases gastro-intestinales est sombre en raison du caractère disséminé de la maladie.

## Conclusion

Les métastases gastro-intestinales du cancer du sein sont très rares; la présence de symptômes gastro-intestinaux chez une malade ayant un antécédent de cancer du sein doit faire suspecter une atteinte métastatique gastro-intestinale.
